# 5FU/Oxaliplatin-Induced Jagged1 Cleavage Counteracts Apoptosis Induction in Colorectal Cancer: A Novel Mechanism of Intrinsic Drug Resistance

**DOI:** 10.3389/fonc.2022.918763

**Published:** 2022-07-01

**Authors:** Maria Pelullo, Sabrina Zema, Mariangela De Carolis, Samantha Cialfi, Maria Valeria Giuli, Rocco Palermo, Carlo Capalbo, Giuseppe Giannini, Isabella Screpanti, Saula Checquolo, Diana Bellavia

**Affiliations:** ^1^ CLN2S - Center for Life Nano- & Neuro Science, Istituto Italiano di Tecnologia, Rome, Italy; ^2^ Laboratory of Molecular Pathology, Department of Molecular Medicine, Sapienza University, Rome, Italy; ^3^ Department of Medico-Surgical Sciences and Biotechnology, Sapienza University, Latina, Italy

**Keywords:** CRC, oxaliplatin, 5FU, Jagged1, GSIs, drug resistance

## Abstract

Colorectal cancer (CRC) is characterized by early metastasis, resistance to anti-cancer therapy, and high mortality rate. Despite considerable progress in the development of new treatment options that improved survival benefits in patients with early-stage or advanced CRC, many patients relapse due to the activation of intrinsic or acquired chemoresistance mechanisms. Recently, we reported novel findings about the role of Jagged1 in CRC tumors with Kras signatures. We showed that Jagged1 is a novel proteolytic target of Kras signaling, which induces Jagged1 processing/activation resulting in Jag1-ICD release, which favors tumor development *in vivo*, through a non-canonical mechanism. Herein, we demonstrate that OXP and 5FU cause a strong accumulation of Jag1-ICD oncogene, through ERK1/2 activation, unveiling a surviving subpopulation with an enforced Jag1-ICD expression, presenting the ability to counteract OXP/5FU-induced apoptosis. Remarkably, we also clarify the clinical ineffectiveness of γ-secretase inhibitors (GSIs) in metastatic CRC (mCRC) patients. Indeed, we show that GSI compounds trigger Jag1-ICD release, which promotes cellular growth and EMT processes, functioning as tumor-promoting agents in CRC cells overexpressing Jagged1. We finally demonstrate that Jagged1 silencing in OXP- or 5FU-resistant subpopulations is enough to restore the sensitivity to chemotherapy, confirming that drug sensitivity/resistance is Jag1-ICD-dependent, suggesting Jagged1 as a molecular predictive marker for the outcome of chemotherapy.

## Introduction

Colorectal cancer (CRC) is one of the most common causes of cancer-related death worldwide (1). Many patients become refractory to systemic therapy and develop relapses, and the median overall survival of metastatic CRC (mCRC) patients is over 30 months (2). To date, in patients with stage III CRC, the adjuvant chemotherapy is the standard treatment after surgery and includes fluoropyrimidine (5FU) combined with leucovorin/irinotecan (FOLFIRI regimen) (3) or with leucovorin/oxaliplatin (OXP) (FOLFOX regimen) (4). Alternatively, capecitabine, an oral form of 5FU, may be combined with irinotecan (XELIRI/CAPIRI) (5) or OXP (XELOX/CAPEOX) (6), which are often used in first-line treatment. In a metastatic setting, the standard combination generally includes 5FU/OXP or 5FU/irinotecan with the addition of at least one biologic drug, such as anti-vascular endothelial growth factor (VEGF) and/or anti-epidermal growth factor receptor (EGFR) monoclonal antibodies (7). Despite the important improvement in overall survival of mCRC patients, most of them become refractory to systemic therapy during or after the treatment and develop relapses.

CRC is a biologically heterogeneous disease with different molecular profiles that reflect specific histopathological and clinical information (8). The *Kras* gene is mutated at codons 12 and 13 in ∼50% of patients with mCRC (9) and is considered a predictive biomarker of resistance to anti-EGFR-based therapy. The prognostic effect of Kras in non-metastatic CRC is controversial. However, the PETACC8 trial shows that Kras mutations have a prognostic value (10, 11), and are associated with poor outcome (90%) in patients with microsatellite-stable tumors (12, 13).

Several studies demonstrate that alterations of the Notch pathway contribute to the CRC onset and malignancy, closely associated with the rapid and uncontrolled proliferation of tumors (14). The activation of the Notch receptors (Notch1-4) occurs upon binding to specific ligands (Jagged1-2 and Delta-like1-3-4), expressed on neighboring cells. This occurrence determines sequential proteolytic cleavages, sustained by ADAM and the (PS)/γ-secretase complex, which allow the intracellular domain of Notch to move into the nucleus and to interact with the DNA-binding factor RBP-Jκ, a process that can be inhibited by γ-secretase inhibitors (GSIs). Inappropriate activation of Notch signaling may cause several cancers and GSIs are recognized as potential anticancer drugs, widely used to inhibit Notch activation (15). In CRC, the effects of GSIs have long been debated, being considered as anticancer drugs able to enhance the chemosensitivity to oxaliplatin (16), or agents that present the ability to counteract oxaliplatin-induced apoptosis (17). Among GSIs, RO4929097, a potent oral inhibitor of γ-secretase, was tested in a phase II study conducted in patients with metastatic, refractory CRC. Consistent with previous reports, the study did not demonstrate any evidence of RO4929097 clinical activity in CRC patients (18, 19).

We have previously demonstrated that the Notch-ligand Jagged1 is directly involved in CRC progression. We have identified the Kras/Erk/ADAM17/Jagged1 signaling axis, able to induce sequential cleavages in the overexpressed Jagged1 protein, mediated by ADAM17 and the PS/γ-secretase complex, resulting in the release of the Jagged1 intracellular domain (Jag1-ICD), which triggers a signaling inside the Jagged1-expressing cells. The Jag1-ICD fragment moves into the nucleus, where it leads to deregulated events, sustaining proliferation, epithelial-to-mesenchymal transition (EMT), invasion/migration, and drug resistance *in vivo* (20). This process occurs when Kras/Erk/ADAM17 signaling is switched on, demonstrating that Jagged1 is a novel proteolytic target of the Kras signaling pathway. We demonstrated that Kras-induced Jagged1 processing is a critical event able to convert the proto-oncogene Jagged1 full length (Jag1-FL) in a novel Jag1-ICD oncogene, whose function plays an important role in sustaining CRC tumor progression.

Herein, we evaluate the effects of GSIs, OXP and 5FU, alone or in combination, on Jagged1 processing in CRC cell lines overexpressing Jagged1. Firstly, we show that GSIs behave as promoting agents, triggering an enforced Jagged1 processing, associated with an increased cellular growth and EMT that confers metastatic properties to cancer cells, in a Notch-independent manner. Moreover, we demonstrate that the most potent anticancer drugs, OXP and 5FU, lead directly to a massive Jag1-ICD activation that results in the selection of a drug-resistant subpopulation. The mechanism of resistance to chemotherapeutic agents is induced by a forced Jag1-ICD accumulation that protects overexpressing Jagged1 CRC cells from apoptosis, under the activation of Jag1-ICD-dependent pro-survival targets. Finally, we provide evidence about synergistic effects induced by GSIs with OXP or 5FU chemotherapeutic agents, in sustaining Jag1-ICD-dependent multidrug resistance, unveiling a novel mechanism of intrinsic chemoresistance in Jagged1 CRC cells, where Jag1-ICD may function as a nuclear effector.

Overall, our data show that Jagged1 processing is directly activated by OXA/5FU chemotherapeutic agents or by GSI compounds, resulting in the release of the oncogene Jag1-ICD, suggesting Jagged1 overexpression as a new potential predictive biomarker, which is useful to predict drug resistance to current therapies and disease recurrence.

## Materials and Methods

### Cell Lines and Treatments

The following human colon cell lines HT29, HCT15, DLD1, LoVo, and SW948 were purchased from the American Type Culture Collection (ATCC) and cultured in opportune medium supplemented with 1% Glutamine (ECB3000D, Euroclone), 1% Antibiotics (ECB3001D, Euroclone), and 10% FBS (Heat-Inactivated; Life Technologies). The media were renewed 3 times per week. All cell lines were cultured at 37°C and with 5% CO_2_. Cells recovered from frozen aliquots were allowed one passage to reach exponential growth phase following recovery before being used. Cells at passages greater than 10 were not used. All cell lines were subjected to routine cell line quality controls (e.g., morphology, *Mycoplasma* #G238, Abm Inc.) and authenticated by DNA profiling [short tandem repeat (STR)] by the cell bank prior to shipping.

An opportune amount of cells was treated with different compounds: DAPT (#65770, Calbiochem), RO4929097 (#S1575, Sigma-Aldrich), LY411575 (#SML0506, Sigma-Aldrich), Semagacestat (#SML1938, Sigma-Aldrich), PF03083014 (#PZ0298, Sigma-Aldrich), U0126 (#662005, Calbiochem), 5-fluorouracil (5FU; #F6627, Sigma-Aldrich), Oxaliplatin (OXP, #O9512, Sigma-Aldrich), and Tapi-2 (#INH-3852-PI).

In order to select resistant cells to OXP and 5FU (HCT15 OXP-R, HCT15 5FU-R, DLD1 OXP-R, and DLD1 5FU-R), HCT15 and DLD1 cells were treated daily for more than 4 weeks with fresh medium containing a low dose of each chemotherapeutic agent (0.5 pmol/μl) (21).

### Cell Viability Assay

CRC cell lines were seeded in 12-well plates at 1 × 10^6^ cells/ml and treated with different GSIs as indicated in figures. DMSO was used as a control vehicle. To perform cell counting, we diluted the cellular suspension 1:2 with Trypan blue stain (T8154, Sigma-Aldrich) and, by using an upright microscope and a Neubauer chamber, we easily counted the living cells and excluded the dead ones. The growth of drug-treated cells was graphed relative to control untreated cells. Measurements were performed in technical triplicates and figures show the averages ± SD of at least 3 biological replicates (22, 23).

### Colony Formation Assay

HCT-15, DLD-1, HCT15 OXP-R, HCT15 5FU-R, DLD1 OXP-R, and DLD1 5FU-R cells were seeded in the appropriate density in 6-well plates. After 24 h, CRC cells were treated with (0.5 pmol/μl) OXA and 5FU, and fresh medium containing drugs was replaced every 2 or 3 days. Twenty-one days after seeding, colonies were visualized by fixing the cells with a mixture of 90% methanol and 10% chloroform, at room temperature for 10 min. Then, they were stained using a solution of 0.1% crystal violet (#HT90132, Sigma-Aldrich) diluted in methanol for 3 min. After staining, plates were washed with water and left to dry overnight. Finally, plates were scanned and stored.

### Cytofluorimetric Analysis

A total of 1 × 10^6^ cells were treated with GSI compounds, OXA and 5FU chemotherapeutic agents, as indicated in figures. Cells were fixed for 30 min in EtOH 70%, washed with PBS, and treated with 100 μg/ml RNase A (cat. #R6513, Sigma-Aldrich) for 15 min. Then, cells were incubated with 10 μg/ml propidium iodide (cat.#P4170, Life Technologies). In order to evaluate apoptosis, cells were fixed and stained with propidium iodide and APC-Annexin V (# 550475, BD Biosciences), following the manufacturer’s instructions. The stained cells were analyzed on a FACS-Calibur with the CellQuest software (24).

### Protein Extracts, Subcellular Fractioning, and Immunoblotting

Whole-cell extract (WCE), subcellular fractioning, and immunoblot assay (25) with the described antibodies (**Supplementary Table S2**) were performed as described elsewhere. Bound antibodies were detected with enhanced chemiluminescence (WesternBright ECL HRP substrate, Advansta Inc.)

### RT-PCR/qRT-PCR

Total RNA extraction and reverse transcription-PCR (RT-PCR) were previously described (26). One microgram of RNA was processed for RT-PCR using the SensiFAST cDNA Synthesis Kit (Bioline). Analysis of gene expression was realized by qPCR using Taq-Man designed assays (**Supplementary Table S1**; Dharmacon Inc.) on the StepOnePlus Real-Time PCR System (Applied Biosystems, Life Technologies), following the manufacturer’s protocol for the comparative *C*_t_ method. Data were analyzed by the ΔΔ*C*_t_ method and *GAPDH* was used for normalization (27).

### RNA Interference Analysis

RNA silencing was performed using 100 nmol/L of *Jagged1* (cat. #L-011060-00-0005) ON-TARGET plus SMART pool small interference RNA (siRNA) or scrambled (cat. #D-001810-10-20, Dharmacon Inc.), using Lipofectamine RNAiMAX (Life Technologies), according to the manufacturer’s instructions (28).

### Wound-Healing Assays

Cell migration was analyzed by wound-healing assay. Briefly, an opportune amount of cells were grown in 6-well plates. Wound injury was made with the tip of a sterile micropipette and cells were allowed to migrate for up to 72 h and photographed (20).

### Statistical Analysis

All results were confirmed in at least three independent experiments and all quantitative data were reported as the mean ± SD. Student’s *t*-tests for unpaired samples were used to assess differences among two groups, while analysis of variance (ANOVA) was used to compare the means among three or more groups. A *p*-value of <0.05 was considered statistically significant (n.s., nonsignificant, *p* > 0.05; *p < 0.5; **p < 0.05; ***p < 0.005, ****p < 0.0005).

## Results

### GSIs Behave as Pro-Tumoral Drugs in CRC Cell Lines Overexpressing Jagged1 by Inducing Jagged1 Processing

**Figure 1:**
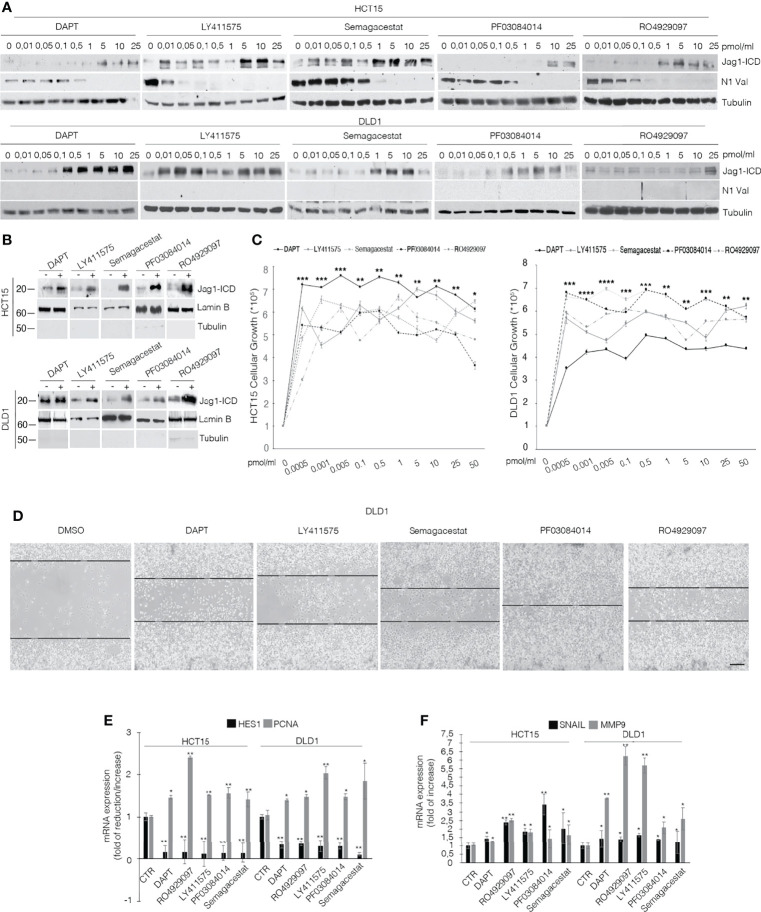
GSIs induce Jagged1 cleavage in Jagged1/KRAS CRC cell lines and induce proliferation and EMT. **(A)** HCT15 and DLD1 cell lines were treated with several GSI compounds (DAPT, LY411575, Semagacestat, PF03084014, and RO4929097) or vehicle alone (DMSO) with the indicated doses (pmol/μl) for 24 h. Whole-cell extracts (WCEs) were subjected to Western blot assay, using indicated antibodies against the Jagged1-intracellular domain (Jag1-ICD) and active Notch1 Valin 1744 (N1Val). Tubulin was used as loading control. **(B)** The subcellular nuclear lysates from HCT15 and DLD1 cells were collected after 48 h of treatment with different GSIs at 10 pmol/μl and immunoblotted as indicated. Protein levels were normalized using Lamin B and Tubulin, as nuclear and cytoplasmatic control, respectively. **(C)** HCT15 and DLD1 cells were treated with increasing doses (pmol/μl) of different GSIs. After 24 h, trypan blue viable cell count was performed to determine the growth rate. All data are representative of at least three independent experiments, each in triplicate. *p < 0.05; **p < 0.05; ***p < 0.005; ****p < 0.0005 (ANOVA test). **(D)** Representative area for wound-healing assay of DLD1 cells after 48 h of scratch, treated with several GSIs. Scale bar, 200 μm. **(E)** *Hes1* and *PCNA* mRNA expression levels were determined by qRT-PCR from GSI-treated cells and normalized relative to human *GAPDH*. Graph was depicted as fold change compared with DMSO-treated cells. Data were presented as mean ± SD. **(F)** qRT-PCR analysis of *mmp9* and *snail* mRNA from CRC cells, treated with different GSIs, was represented as fold changes ± SD after intrasample normalization to the level of *GAPDH.* All data are representative of at least three independent experiments, each in triplicate. *p < 0.05; **p < 0.05; ***p < 0.005 (Student’s *t-*test).

To discriminate the biological outcome of Jag1-ICD on CRC chemoresistance, we firstly investigated the effects of GSIs on Jagged1 processing. Several Kras CRC cell lines (**Supplementary Figure 1A**) expressing Notch and Jagged1 at different levels (**Supplementary Figure 1B**) were treated with various GSI compounds. As expected, GSIs inhibit Notch1 cleavage but, unexpectedly, they induce an enforced Jag1-ICD release, according to dose (**Figure 1A**; **Supplementary Figure 1C**), favoring its nuclear localization (**Figure 1B**; **Supplementary Figure 1D**), which is associated with important biological effects, such as increased cellular proliferation (**Figure 1C**) and higher cell migration (**Figure 1D**). Significantly, the GSI-dependent Jag1-ICD accumulation empowers the activation of genes involved in proliferation, EMT, and invasion, such as *PCNA*, *Snail*, and *MMP9*, sustaining higher cell migration (**Figures 1E, F**) (20), in a Notch-independent manner, as confirmed by *Hes1* decreased expression (**Figure 1E**).

Previously, we established that Kras driver mutation triggers the MEK/Eryk/ADAM17 signaling axis that results in Jagged1 processing, through Erk activation (20). Since GSIs have the ability to induce Erk activation (16), we hypothesized that they induce Jagged1 processing through the Erk pathway. **Supplementary Figure 2A** shows that GSI compounds induce a rapid release of the Jag1-ICD fragment, strictly linked to Erk activation in CRC cells. Accordingly, the MEK inhibitor U0126 abrogates Jagged1 processing and the addition of GSIs restores the Jag1-ICD accumulation, after washing out of Erk inhibition (**Supplementary Figure 2B**).

Altogether, these data strongly suggest the pro-tumoral effects of GSI compounds, through reinforcing Jagged1 processing *via* the MAPK pathway, in CRC cell lines overexpressing Jagged1 full-length.

### Jagged1 Activation Addresses CRC Cells Towards Intrinsic Drug Resistance to OXP and 5FU

**Figure 2:**
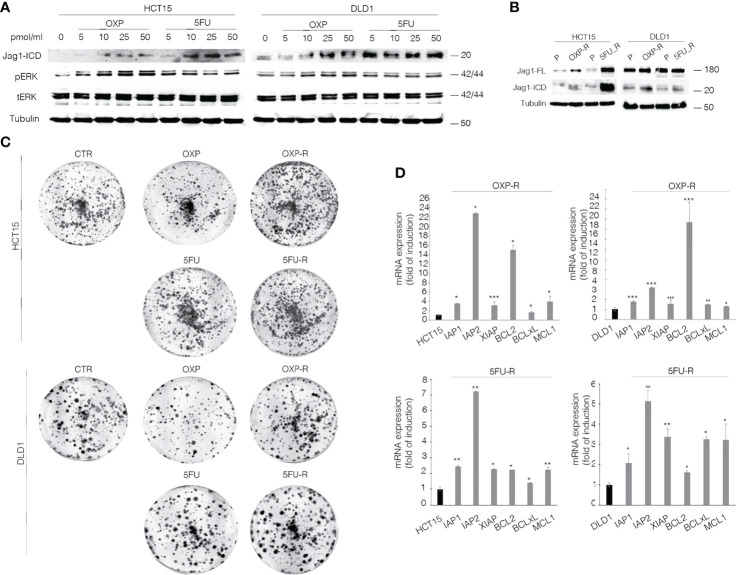
OXP and 5-FU induce Jag1-ICD accumulation that drives CRC cells towards intrinsic drug resistance against the common chemotherapeutic agents. **(A)** Whole-cell extract from HCT15 and DLD1 cells, treated with OXP and 5FU with increasing doses (5–10–25–50 pmol/μl) for 24 h, was subjected to immunoblotting analysis. **(B)** Parental (P), OXP-R, and 5FU-R CRC cells, were collected and subjected to immunoblotting assays as indicated. Tubulin was used as loading control. **(C)** Colony-forming ability of HCT-15 and DLD-1 cells or OXP-R and 5FU-R HCT-15 and DLD-1 cells in the presence of OXA and 5FU, compared with vehicle alone. A total of 2 × 10^3^ cells were seeded in 6-well plates and treated with 0.5 pmol/μl of OXP or 5FU for 15 days. **(D)** qRT-PCR analysis of *iap1, iap2, xiap, bcl2, bclXl*, and *mcl1* mRNAs in parental (P), OXP-R, and 5FU-R CRC cells was performed. Data are reported as fold changes ± SD after intrasample normalization to the level of *GAPDH*. All data are representative of at least three independent experiments, each in triplicate. *p < 0.05; **p < 0.01; ***p < 0.001 (Student’s *t*-test).

To unequivocally demonstrate the functional role of the Jag1-ICD signaling in CRC drug resistance, we firstly analyzed Jagged1 processing in HCT15 and DLD1 cells upon OXP and 5FU treatments. **Figure 2A** shows that chemotherapeutic agents induce a strong Jag1-ICD accumulation in a dose-dependent manner, associated with Erk activation. Oxaliplatin-resistant (OXP-R) and 5FU-resistant (5FU-R) cell lines were derived from exposure to chronic low dose of OXP and 5FU for 4 weeks. Remarkably, OXP-R and 5FU-R cells express a massive accumulation of Jag1-ICD compared to parental cells (P) (**Figure 2B**). Interestingly, OXP-R and 5FU-R subpopulations treated with both drugs show an increased ability to support long-term survival measured in a colony-forming assay, compared to parental cells (**Figure 2C**). To investigate resistance mechanism induced by Jag1-ICD, we analyzed genes directly involved in apoptotic signaling cascade. OXP-R and 5FU-R cells present a strong upregulation of *c-IAP1, c-IAP2*, and *XIAP* transcripts; inhibitors of the Caspase activity; and *BCL-2, BCL-xL*, and *MCL1* genes, belonging to anti-apoptotic BCL-2 family members, compared with P cells (**Figure 2D**).

Our data demonstrate that CRC cells overexpressing Jagged1 activate intrinsic chemoresistance mechanisms against OXP and 5FU anti-cancer drugs, based on Jagged1 processing OXP- and 5FU-induced. OXP and 5FU show the ability to directly induce Jag1-ICD release, which sustains increased expression of anti-apoptosis-related genes and counteracts OXP- or 5FU-induced cytotoxic effects. Therefore, Jag1-ICD exerts a cytoprotective effect on Jagged1 CRC cells, promoting tumoral cells to escape from cancer drugs and sustaining the chemoresistance.

### GSI-Mediated Jag1-ICD Accumulation Promotes Cell Cycle Progression in Surviving OXP- and 5FU-Treated Cells

**Figure 3:**
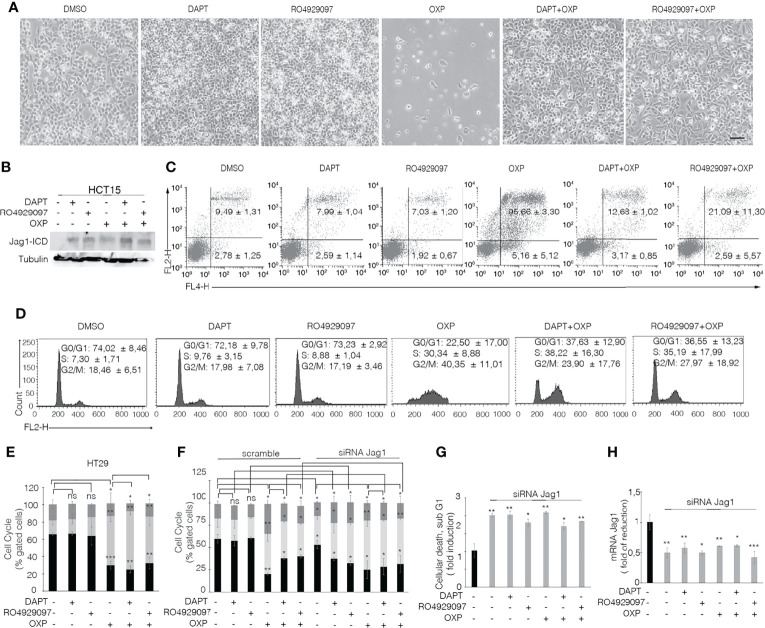
GSIs operate as promoting agents in Jagged1/KRAS CRC cells and help cells escape from the OXP-induced cell cycle block in a Jag1-ICD-dependent manner. **(A)** Representative plate area of HCT15 cells treated with 10 pmol/μl of DAPT, RO4929097, and OXP alone or in combination. Scale bar, 200 μm. **(B)** An amount of HCT-15 cells from panel A were collected and whole-cell extracts were immunoblotted or **(C)** stained against Annexin V (APC) with/without propidium iodide (PI) to evaluate the percentage of apoptotic cells and **(D)** cell cycle. **(E)** HT29 cells treated for 24 h with 10 pmol/μl of DAPT, RO4929097, and OXP alone or in combination were stained with propidium iodide (PI) to analyze cell cycle progression. **(F)** Jagged1-silenced HCT15 cells (siRNA-Jagged1) or control siRNA (scramble) were treated for 24 h with 10 pmol/μl DAPT, RO4929097, and OXP, alone or in combination, and stained with propidium iodide (PI) to analyze cell cycle progression and **(G)** subG1 cellular death. **(H)** *Jag1* mRNA expression levels revealed by qRT-PCR assay in Jagged1-silenced HCT15 cells or scramble cells, from panel **(F)** Results are expressed as fold of reduction relative to control. Data were normalized with respect to GAPDH expression. All data are representative of at least three independent experiments, each in triplicate. ns, *p* > 0.5; *p < 0.05; **p < 0.01; ***p < 0.001 (Student’s *t*-test).

**Figure 4:**
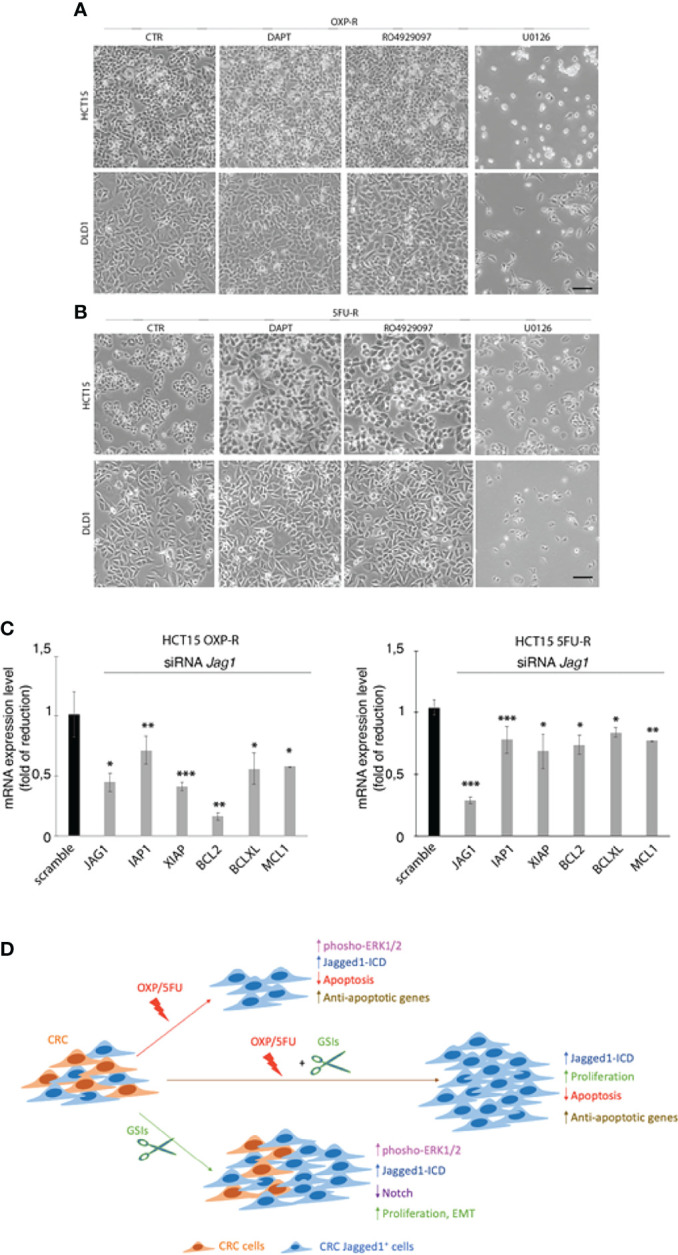
The inhibition of Jagged1 signaling sensitizes the CRC cells to chemotherapy. **(A)** Representative plate area of OXP-R and **(B)** 5FU-R CRC cells, treated for 24 h with 10 pmol/μl of DAPT and RO4929097 and 30 pmol/μl of U0126. Scale bar, 200 μm. **(C)** The expression analysis of *iap1, iap2, xiap, bcl2, bclXl*, and *mcl1* mRNAs was performed by qRT-PCR in OXP-R and 5FU-R HCT15 cells, silenced for *Jagged1*. Data are reported as fold changes ± SD after intrasample normalization to the level of *GAPDH*. All data are representative of at least three independent experiments, each in triplicate. *p < 0.05; **p < 0.01; ***p < 0.001 (Student’s *t*-test). **(D)** The cartoon summarizes the role of Jagged1 in chemoresistance. In detail, GSIs (green arrow) inhibit Notch signaling, but strongly trigger the Jagged1 reverse signaling by activating the ERKs cascade that induces a huge accumulation of Jag1-ICD. GSIs, *via* Jag1-ICD, sustain the proliferation and migration/invasion of CRC cell lines. Similarly, the chemotherapeutics agents OXP/5FU (red arrow) lead to Jagged1 processing, through the activation of ERKs. Jag1-ICD, induced by OXP/5FU, enhances the expression of several anti-apoptotic genes, supporting the growth of a chemoresistant Jagged1-positive subpopulation (OXP-R and 5FU-R). Finally, the combination of GSIs and OXP/5FU (brown arrow) sustains the Jag1-ICD-dependent signaling and reinforces the activation of pro-survival and anti-apoptotic events, unveiling the role of Jag1-ICD in mechanisms of chemoresistance.

To determine that GSIs are able to empower CRC proliferation and drug resistance by inducing Jagged1 processing, we firstly treated HCT15 and DLD1 cells with GSIs and OXP alone or in combination. Interestingly, GSIs have no effect on viability, but they promote cellular growth, when compared to vehicle alone (DMSO); conversely, platinum compound alone strongly leads to a massive cellular death and to selection of a surviving resistant subpopulation expressing Jag1-ICD (**Figures 3A, B**). Interestingly, Jag1-ICD counteracts oxaliplatin-induced death, and the addition of GSIs to oxaliplatin helps cells escape from G2/M cell cycle arrest, when compared to OXP treatment, resulting in the rapid increase of cells expressing Jag1-ICD (**Figures 3B–D**). To provide additional evidence that GSI effects on cell cycle progression are mediated by Jag1-ICD accumulation, we performed FACS analysis in both HT29 (**Figure 3E**), a CRC cell line not expressing Jagged1 (**Supplementary Figure 1B**), and the *Jag1*-silenced HCT15 cell line (**Figures 3F–H**) upon GSIs and OXP treatment, alone or in combination. Upon OXP treatment, cell cycle analysis revealed that the absence of Jagged1 allows a severe cell cycle block, resulting in OXP-induced cellular apoptosis. Moreover, the addition of GSIs to OXP is not able to abrogate the oxaliplatin-dependent cytotoxic effects, neither in HT29 nor in Jagged1 knockdown HCT15 cells, which display a persistent cell cycle blockage. Similarly, GSI-induced cell cycle progression is also observed in Jagged1-overexpressing CRC cells upon 5FU-treatment (**Supplementary Figure 3**). To unequivocally demonstrate the function of Jag1-ICD in drug resistance, OXP-R and 5FU-R CRC cell lines were treated with GSIs with no effect on viability (**Figures 4A, B**); conversely, the U0126 compound, which is able to inhibit Erk activation, rapidly counteracts Jagged1 processing (**Supplementary Figures 4A, B**), increasing chemosensitivity to OXP and 5FU (**Figures 4A, B**). Accordingly, the siRNA-mediated depletion of *Jagged1* in OXP-R and 5FU-R subpopulations (**Supplementary Figures 4C, D**) determines a drastic downregulation of anti-apoptotic genes, with respect to control cells (**Figure 4C**).

Overall, our data indicate that GSIs may behave as promoting agents in CRC cells with Jagged1 overexpression. In addition, we demonstrate that the Jagged1 silencing or the abrogation of Jagged1 cleavage by TAPI2 is necessary to overcome the drug resistance.

## Discussion

Chemotherapy resistance in CRC patients remains a critical clinical challenge since it allows development of metastasis and disease recurrence. CRC presents a complex molecular heterogeneity that is not yet fully known. Likewise, the molecular mechanisms that characterize CRC drug resistance are not well understood and novel biomarkers predicting therapy response must be identified (8). To date, only *Kras* mutation has a predictive value of poor response to anti-EGFR monoclonal antibodies. Therefore, understanding molecular mechanisms that lead to cancer drug resistance is a key point for finding novel therapeutic approaches. The standard care for CRC patients is commonly based on the combination of chemotherapy drugs that includes fluorouracil and leucovorin (LV), which work together to inhibit DNA/RNA synthesis and to modulate tumor growth. Observational studies report conflicting findings about oxaliplatin survival benefits as part of the standard care for the adjuvant treatment of patients with early-stage CRC (29, 30). Also in advanced colon cancer, the addition of OXP to 5FU with LV (FOLFOX), administered to patients as first-line treatment, significantly improves antitumor efficacy only for a few months after treatment (31). Then, a large part of patients stop responding to chemotherapeutic agents, due to intrinsic and acquired drug resistance, whose mechanisms include the activation of the Erk pathway that cross-talks with oncogenic signaling pathways, converging on the regulation of apoptosis pathways (32). An attempt to conduct molecularly targeted therapy was made using GSIs, which induce the pharmacologic inactivation of Notch signaling. The data on GSIs in CRC, used alone or in combination with chemotherapeutic agents, are quite diverging. Firstly, they were recognized as potential anti-cancer drugs (16), then as compounds with the ability to abrogate OXP-induced apoptosis (17); ultimately, GSIs were tested in clinical trial in patients with refractory mCRC and RO4929097 monotherapy, and demonstrated no evidence of clinical activity. Therefore, the potent GSI RO4929097 was not recommended in this malignancy (18). These observations strongly suggest that the chemoresistance in CRC could be induced by unknown molecular mechanisms, possibly associated with a unique set of molecular changes that make cancer cells resistant to the effects of chemotherapeutic drugs.

Interestingly, recent observations suggest that the Notch-ligand Jagged1 is expressed in an aberrant manner in about 50% of colon tumors, and the higher levels of expression correlate with differentiation parameters and stages of CRC (33–36). Although originally found to play a role as a ligand of canonical Notch signaling, we recently showed that a Notch-independent Jagged1 reverse signaling is implicated in the pathogenesis of CRC (20). It is known that the aberrant APC/β-catenin signaling is directly responsible for Jagged1 overexpression, required for tumorigenesis in the intestine (20, 37, 38). In addition, we identified the constitutive activation of the Kras/Erk/ADAM17 signaling axis, which triggers PS/γ-secretase complex activation, able to induce sequential cleavages in the Jagged1 protein, lastly resulting in the release of the Jagged1 intracellular fragment (Jag1-ICD), which sustains reverse intracellular signaling. This process occurs when the Kras/Erk/ADAM17 axis is switched on, demonstrating that Jagged1 is a novel proteolytic target of Kras signaling. We demonstrated that the constitutive processing of the Jagged1 protein is a critical event able to convert the proto-oncogene Jag1-FL in a novel Jag1-ICD oncogene, whose function plays an important role in sustaining tumor progression *in vivo* (20).

In the present study, we have evaluated the effects of OXP and 5FU chemotherapeutic agents, alone or in combination with GSIs, on Jagged1 processing/activation in CRC cells. First, we show that GSIs are able to inhibit Notch signaling, as expected, but surprisingly they lead to a strong activation of Jagged1 reverse signaling through Erk1/2 activation, resulting in an enforced Jag1-ICD release with oncogenic function, in a Notch-independent manner. GSIs do not disturb CRC viability but induce cellular growth functioning as tumor-promoting agents through Jagged1 processing. Here, we also demonstrate that OXP and 5FU, the most potent anticancer drugs, lead to a strong Jagged1 processing *via* Erk1/2 activation, which induces mechanisms of intrinsic chemoresistance. The resistance to OXP/5FU drugs protects Jagged1-overexpressing CRC cells from apoptosis through the activation of Jag1-ICD-dependent pro-survival targets, also under genotoxic stress induced by chemotherapeutic agents. Finally, we provide evidence about the synergistic effects induced by GSIs with OXP or 5FU chemotherapeutic agents, in sustaining Jag-ICD-dependent multidrug resistance, unveiling a novel mechanism of drug resistance in Jagged1 CRC cells, where Jag1-ICD may function as a nuclear effector with the ability to sustain the pro-proliferative and anti-apoptotic events (**Figure 4D**).

Overall, our data point out Jagged1 overexpression as a new potential predictive biomarker that is useful in predicting cancer progression and drug resistance to current therapies for early and advanced CRC favoring tumor relapse.

## Data Availability Statement

The raw data supporting the conclusions of this article will be made available by the authors, without undue reservation.

## Author Contributions

MP and DB conceived, designed and supervised the research; MP and SZ performed the experiments; MP, SZ, RP, CC, SChe and DB analyzed and interpretated the data; GG, IS, SChe and DB wrote and reviewed the manuscript; SCi, MDC, MVG provided administrative, technical and /or material support. All the authors reviewed the data and approved the final version of the manuscript.

## Funding

This work was supported by the following grants: Sapienza University 2018 project #RP1181643121DD86 (to DB), Sapienza University 2020 Project No: RG120172B8354E7F (to DB), and MIUR PNR 2015-2020 ARS01_00432, PROGEMA (to IS).

## Conflict of Interest

The authors declare that the research was conducted in the absence of any commercial or financial relationships that could be construed as a potential conflict of interest.

## Publisher’s Note

All claims expressed in this article are solely those of the authors and do not necessarily represent those of their affiliated organizations, or those of the publisher, the editors and the reviewers. Any product that may be evaluated in this article, or claim that may be made by its manufacturer, is not guaranteed or endorsed by the publisher.
