# Supplementary material for: 5FU/Oxaliplatin-Induced Jagged1 Cleavage Counteracts Apoptosis Induction in Colorectal Cancer: A Novel Mechanism of Intrinsic Drug Resistance
Source: Front Oncol. 2022 Jul 1;12:918763. doi: 10.3389/fonc.2022.918763 (PMC9283835; doi:10.3389/fonc.2022.918763)
Supplement: Supplementary file 1 [file DataSheet_1.pdf]

Supplemental Figures

5FU/Oxaliplatin-induced Jagged1 cleavage counteracts apoptosis induction in colorectal cancer. A novel mechanism of intrinsic drug-resistance.

Maria Pelullo, Sabrina Zema, Mariangela De Carolis, Samantha Cialfi, Maria Valeria Giuli, Rocco Palermo, Carlo Capalbo, Giuseppe Giannini, Isabella Screpanti, Saula Checquolo and Diana Bellavia

Supplemental Figure 1. GSIs sustain the Jagged1 cleavage in CRC cell lines.

Supplemental Figure 2. GSIs induce Jagged1 processing by activating ERK signaling cascade, in CRC.

Supplemental Figure 3. The Jag1-ICD induced by GSIs intensifies the CRC resistance against 5-Fluoraracil.

Supplemental Figure 4. The abrogation of Jagged1 signaling in CRC chemoresistant cells.

Supplemental Table 1. List of primers utilized in this study

Supplemental Table 2. List of antibodies utilized in this study

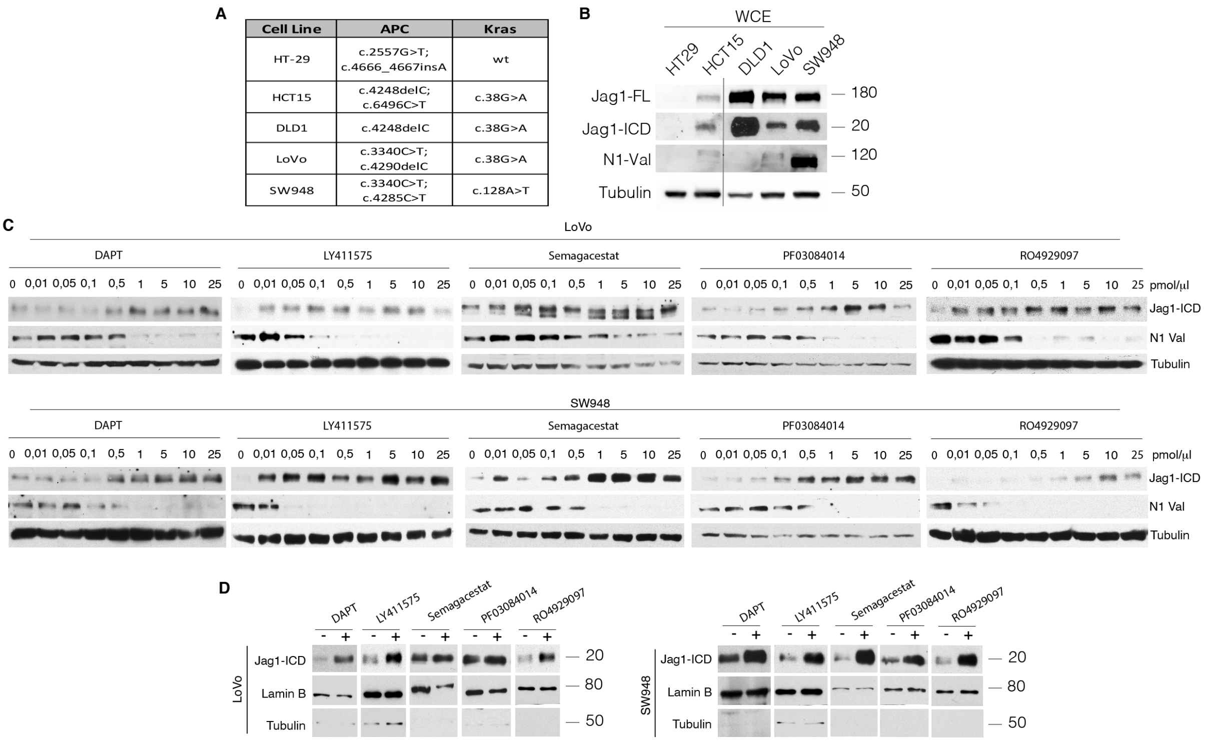

Cell Line	APC	Kras
HT-29	c.2557G>T; c.4666_4667insA	wt
HCT15	c.4248delC; c.6496C>T	c.38G>A
DLD1	c.4248delC	c.38G>A
LoVo	c.3340C>T; c.4290delC	c.38G>A
SW948	c.3340C>T; c.4285C>T	c.128A>T

Supplemental Figure 1.

GSIs sustain the Jagged1 cleavage in CRC cell lines. **A,** The table shows the APC and KRAS mutation status in HT29, HCT15, DLD1, LoVo and SW948 cell lines. **B,** Characterization of Jagged1 and Notch signaling in several CRC cell lines. The whole cell extract of HT29, HCT15, DLD1, LoVo and SW948 cells were immunoblotted for Jag1 full-length (Jag1-FL), Jag1 intracellular domain (Jag1-ICD), Notch1-Val1774. Tubulin was used as loading control. **C,** GSIs activate the Jag1 retrograde signalling. The LoVo and SW948 cell lines were treated for 24 hours with the indicated doses (pmol/µl) of different GSIs (DAPT, LY411575, Semagacestat, PF03084014, RO4929097) or DMSO and subjected to Western blot assay against the specified antibodies. Tubulin was used as loading control. **D,** The nuclear localization of Jag1-ICD is still maintained upon GSIs treatment. The subcellular fractioned lysates of LoVo and SW948 cells, collected after 48 hours of treatment with a fix dose (10 pmol/µl) of different GSIs (DAPT, LY411575, Semagacestat, PF03084014, RO4929097), were subjected to Western blot assay as indicated. Protein levels were normalized relative to Lamin B and Tubulin as nuclear and cytoplasmatic control, respectively. All data are representative of at least three independent experiments.

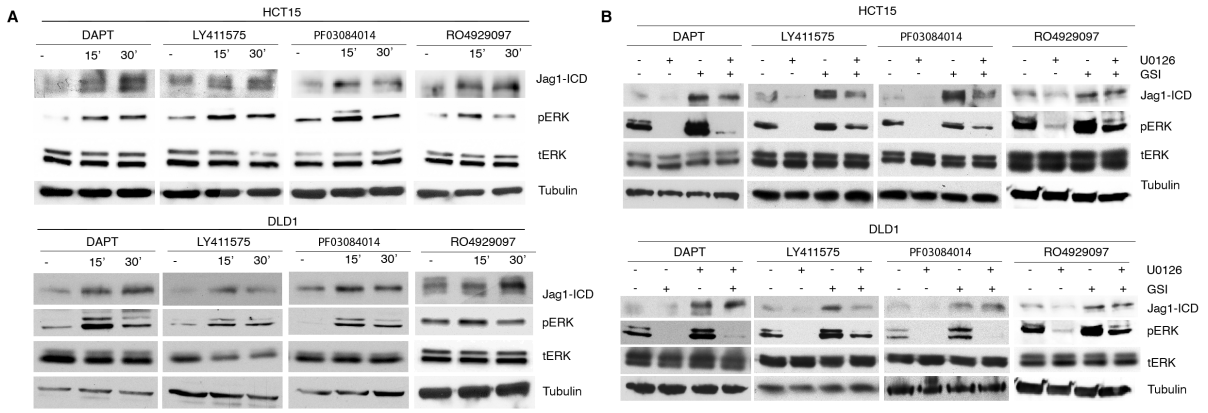

Supplemental Figure 2.
GSIs induce Jagged1 processing by activating ERK signaling cascade, in CRC. **A,** The GSIs quickly activate the ERKs cascade. HCT15 (upper panels) and DLD1 (lower panels) were treated for 15 and 30 minutes with a fix dose (10 pmol/µl) of different GSIs (DAPT, LY411575, Semagacestat, PF03084014, RO4929097). The harvested cells were lysate and subjected to Western blot assay against Jag1-ICD, pERK and totERK. **B,** GSIs restore the intracellular accumulation of Jag1-ICD after washing-out of ERKs-inhibition. The whole-cell extract (WCE) derived from HCT15 and DLD1 cell lines, treated with 10 pmol/µl of different GSIs (DAPT, LY411575, Semagacestat, PF03084014, RO4929097) and with 30 pmol/µl of U0126 alone or in combination, were analysed by Western blotting assays for the indicated antibodies. The protein levels normalized respective to Tubulin. All data are representative of at least three independent experiments.

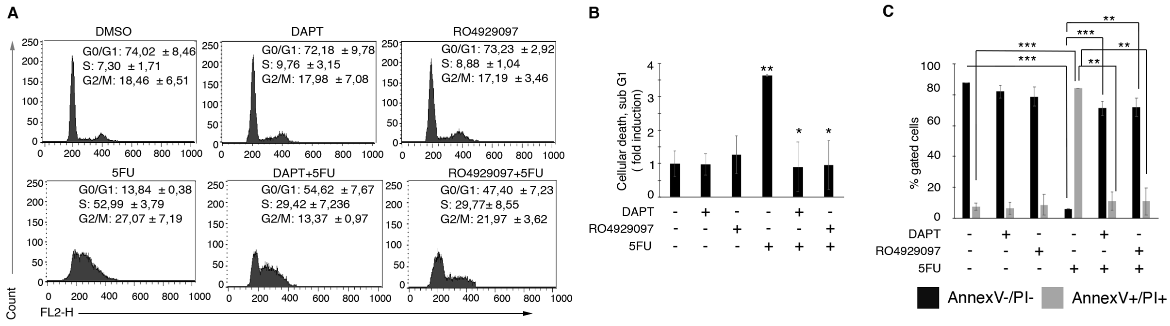

Supplemental Figure 3.

The Jag1-ICD induced by GSIs intensifies the CRC resistance against 5-Fluoraracil. **A-C,** GSIs abrogate the 5FU-dependent cell cycle block and death. The HCT15 cells treated for 24 hours with 10 pmol/µl of DAPT, RO4929097 and 5FU alone or in combination were fixed and stained with Propidium Iodide (PI) and/or Annexin V (APC) to analyse the cell cycle progression (A), the subG1 cellular death (B) and the apoptotic rate (C). All data are representative of at least three independent experiments, each in triplicate. *, $P < 0.05$; **, $P < 0.01$; ***, $P < 0.001$ (Student *t* test).

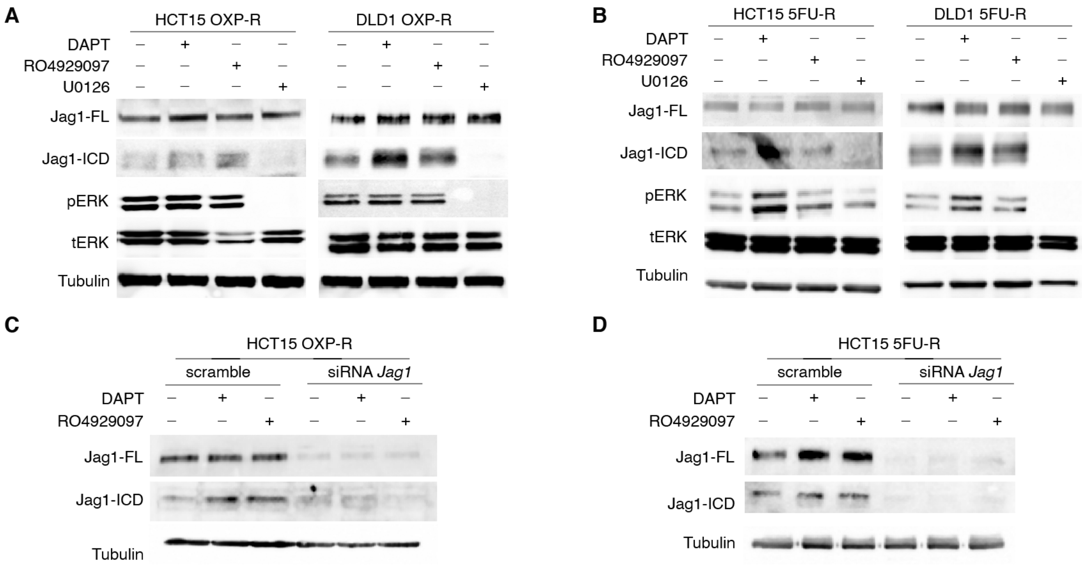

Supplemental Figure 4.
The abrogation of Jagged1 signaling in CRC chemoresistant cells. **A and B,** HCT15 and DLD1 OXP-Resistant (OXP-R, A) and 5FU-Resistant (5FU-R, B) cells, treated for 24 hours with 10 pmol/µl of DAPT and RO4929097 and with 30 pmol/µl of U0126, were collected. The lysates were subjected to Western blot assay for the indicated antibodies: Jag1-ICD, pERK, tERK. **C and D,** The whole cell extracts of HCT15 OXP-Resistant (OXP-R, C) and 5FU-Resistant (5FU-R, D) cells, transfected for 24 hours with siRNA specific for Jagged1 (siRNA Jag1) or with a non-specific siRNA (scramble) and treated with 10 pmol/µl of DAPT and RO4929097 for 24 hours, were immunoblotted against the specified antibodies. Tubulin was used as loading control.

Gene	Taqman Ref
Jagged1	Hs01070032_m1
Hes1	Hs00172878_m1
PCNA	Hs00427214_g1
MMP9	Hs00234579_m1
Snail	Hs00195591_m1
IAP1	Hs01112284_m1
IAP2	Hs00985031_g1
XIAP	Hs00745222_s1
Survivin	Hs00153353_m1
BCL2	Hs04986394_s1
BCLXL	Hs00236329_m1
MCL1	Hs06626047_g1
FLIP	Hs01116280_m1
GAPDH	Hs02758991_g1

Supplemental Table 1. List of primers utilized in this study

**Primary antibody**	**Source**	**Reference**	**Dilution**
Jagged1	Sigma-Aldrich	HPA021555	1:1000
Notch1 Val1744	Cell Signaling	#4147S	1:1000
Phosho-ERK (pERK)	Santa Cruz Biotchenology	SC-7383	1:1000
Total ERK (tERK)	Cell Signaling	#4695S	1:1000
Caspase 3	Cell Signaling	#9661S	1:1000
Lamin B	Santa Cruz Biotchenology	SC-6217	1:1000
Tubulin	Sigma-Aldrich	#T9026S	1:10000
**Secondary antibody**	**Source**	**Reference**	**Dilution**
Donkey anti rabbit-HRP	Bethyl	A120-108P	1:30000
Goat anti mouse-HRP	Bethyl	A90-116P	1:30000

Supplemental Table 2: List of antibodies utilized in this study
